# A new method for the synthesis of α-aminoalkylidenebisphosphonates and their asymmetric phosphonyl-phosphinyl and phosphonyl-phosphinoyl analogues

**DOI:** 10.3762/bjoc.11.153

**Published:** 2015-08-13

**Authors:** Anna Kuźnik, Roman Mazurkiewicz, Mirosława Grymel, Katarzyna Zielińska, Jakub Adamek, Ewa Chmielewska, Marta Bochno, Sonia Kubica

**Affiliations:** 1 Department of Organic Chemistry, Biochemistry and Biotechnology, Silesian University of Technology, B. Krzywoustego 4, 44-100 Gliwice, Poland; 2 Department of Bioorganic Chemistry, Wrocław University of Technology, Wybrzeże Wyspiańskiego 27, 50-370 Wrocław, Poland

**Keywords:** alkylidenebisphosphonate, α-amino acid phosphorus analogues, electrochemical α-methoxylation, 1-phosphinoylalkylphosphonate, 1-phosphinylalkylphosphonate

## Abstract

A convenient approach has been developed to α-aminoalkylidenebisphosphonates and their asymmetric phosphonyl-phosphinyl and phosphonyl-phosphinoyl analogues by α-phosphonylation, α-phosphinylation or α-phosphinoylation of 1-(*N*-acylamino)alkylphosphonates, that, in turn, are easily accessible from *N*-acyl-α-amino acids. Effective electrophilic activation of the α-position of 1-(*N*-acetylamino)alkylphosphonates was achieved by electrochemical α-methoxylation of these compounds in methanol, mediated with NaCl, followed by displacement of the methoxy group with triphenylphosphonium tetrafluoroborate to give hitherto unknown 1-(*N*-acetylamino)-1-triphenylphosphoniumalkylphosphonate tetrafluoroborates. The latter compounds react smoothly with trialkyl phosphites, dialkyl phosphonites or alkyl phosphinites in the presence of Hünig’s base and methyltriphenylphosphonium iodide in a Michaelis–Arbuzov-like reaction to give the expected alkylidenebisphosphonates, 1-phosphinylalkylphosphonates or 1-phosphinoylalkylphosphonates, respectively, in good yields.

## Introduction

**Figure 1:**
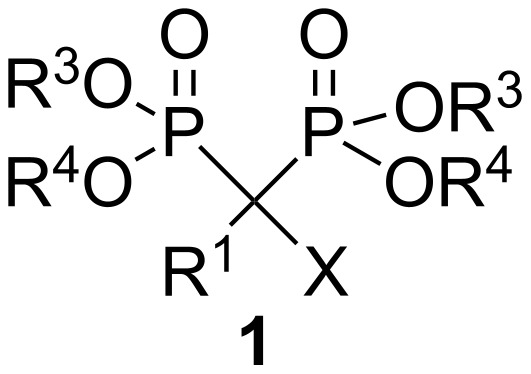
General structure of bisphosphonates.

α-Aminophosphonic and α-aminophosphinic acids, as phosphorus analogues and bioisosters of α-amino acids, exhibit a variety of important biological properties [1–3]. α-Aminobisphosphonates of general formula **1** (Figure 1, X = NHR), that can be considered as one of the most interesting subclass of α-aminophosphonic acids [1,3], are an important class of drugs that are currently successfully being used to treat osteoporosis and similar diseases, including Paget’s disease (*osteitis deformans*), bone metastasis (with or without hypercalcaemia), Kahler’s disease (*multiple myeloma*), primary hyperparathyroidism, brittle bone disease (*osteogenesis imperfecta*), fibrous dysplasia and others [1,4].α‑Aminobisphosphonates (Figure 1, X = NHR) and α‑hydroxybisphosphonates (Figure 1, X = OH) that can easily be obtained from α‑amino derivatives [5–6] belong to two of the most important subclasses of bisphosphonates with many successful medical applications (Table 1) [1,3–4,7–9].

**Table 1:** Antiresorptive potency of selected α-amino and α-hydroxy derivatives of bisphosphonates [3,7–8].

Comp.	Generic name	X	R^1^	R^2^	R^3^	R^4^	ED_50_ [µg/kg]

**1a**	Incadronate	NHR^2^	H	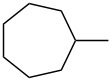	H	Na	7.0
**1b**	NE 58025	NHR^2^	H	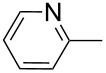	H	H	
**1c**	NE 97220	NHR^2^	H	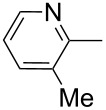	H	H	
**1d**	Risedronate	OH	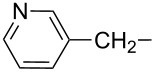	–	H	H	12.0
**1e**	Ibandronate	OH	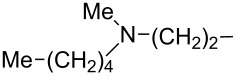	–	H	H	1.1
**1f**	Zoledronate	OH	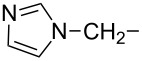	–	H	H	0.07

The most common methods for the synthesis of 1-aminoalkylidenebisphosphonic acid derivatives consist in the consecutive formation of two C_α_–P bonds between a carbon electrophile (most often an electrophilic imine intermediate) and two identical molecules of the proper phosphorus nucleophile. Another group of methods requires the formation of a C_α_–N bond between an easily accessible, symmetrical, nucleophilic methylenebisphosphonic acid derivative and a nitrogen electrophile. Both groups of methods result in symmetrical products with the same phosphonyl or dialkoxyphosphonyl groups [10–18].

**Figure 2:**
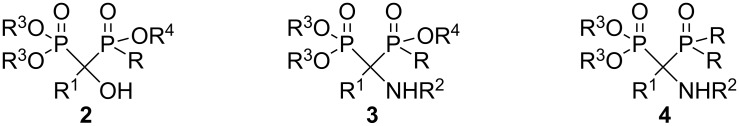
General structures of 1-hydroxy- and 1-amino-1-phosphinylalkylphosphonates (**2** and **3**, respectively) and 1-amino-1-phosphinoylalkylphosphonates **4**.

Up until 1989, very little was known about the preparative feasibility and biological activity of 1-phosphinylalkylphosphonates, including their most interesting 1-hydroxy and 1-amino derivatives (Figure 2, **2** and **3**, respectively; R = H, Alk, Ar). In 1990, Ebetino et al. described both a contiguous and stepwise strategy for the synthesis of the pyridylaminomethane-based class of phosphinylalkylphosphonates **3** (Figure 2, R = Me or *n*-butyl, R^1^ = H, R^2^ = Py and its derivatives, R^3^, R^4^ = H), and discovered the bone resorption-inhibition ability of these compounds [19–21]. They also registered a number of patents relating to methods for treating or preventing diseases characterised by abnormal calcium and phosphate metabolism by utilising 1-phosphinylalkylphosphonic acid derivatives, especially their 1-amino and 1-hydroxy derivatives, and their pharmaceutical compositions [22–27].

In the contiguous method, the condensation of 2-amino-3-picoline with triethyl orthoformate, diethyl phosphite and monoethyl methylphosphonite, followed by chromatographic separation of the three possible condensation products and the final hydrolysis, gave the expected phosphinylalkylphosphonic acid in only 17% yield [20]. The stepwise method consisted in the condensation of diethyl chlorophosphonite with triethyl orthoformate to diethyl 1,1-diethoxymethylphosphonate [(EtO)_2_CHPO(OEt)_2_], followed by condensation of the latter compound with 2-amino-3-picoline and monoethyl methylphosphonite. The final hydrolysis gave the expected phosphinylalkylphosphonic acid in 35% yield based on 2-amino-3-picoline [20].

In 1989, Schrader and Steglich described an example of synthesis of diethyl 1-(*N*-acylamino)-1-[ethoxy(methylphosphinyl)]methylphosphonate using a Michaelis–Arbuzov-like condensation of the corresponding ethyl [1-(*N*-acylamino)-1-bromomethyl]methylphosphinate with triethyl phosphite in 79% yield [28].

The first described synthesis of 1-amino-1-phosphinoylalkylphosphonates **4** (Figure 2), reported by Kostka and Kotyński in 1990–1993 was the synthesis of diethyl 1-(*N*-salicyloylamino)-1-diphenylphosphinoylmethylphosphonate (Figure 2, **4**: R = Ph, R^1^ = H, R^2^ = *o*-HOC_6_H_4_CO, R^3^ = Et) by condensation of diethyl 2,3-dihydro-4*H*-1,3-benzoxazin-4-one-2-phosphonate with diphenylphosphine oxide [29] or diphenyltrimethylsilyloxyphosphine [Ph_2_POSiMe_3_] [30]. In 2004–2007, Onys’ko et al. reported the synthesis of diethyl or diphenyl 1-(*N*-phenylsulfonylamino)-1-diphenylphosphinoylmethylphosphonate derivatives (Figure 2, **4**: R = Ph, R^1^ = Ar or CCl_3_, R^2^ = SO_2_Ph, R^3^ = Et or Ph) and diethyl 1-amino-1-diphenylphosphinoyl-2,2,2-trifluoroethylphosphonate (Figure 2, **4**: R = Ph, R^1^ = CF_3_, R^2^ = H, R^3^ = Et) by the addition of diphenylphosphine oxide to the corresponding arylimidoyl-, trichloroacetimidoyl- or trifluoroacetimidoylphosphonates [R^1^(C=NR^2^)PO(OR^3^)_2_; R^1^ = Ar, CCl_3_ or CF_3_, R^2^ = SO_2_Ph or H, R^3^ = Et or Ph] [31–34]. In 2009, Prishchenko et al. reported an example of synthesis of diethyl 1-dimethylamino-1-(dipropylphosphinoyl)methylphosphonate (Figure 2, **4**: R = Pr, R^1^ = H, R^3^ = Et) by condensation of [1-ethoxy-1-(dimethylamino)methyl]dipropylphosphine oxide [(Pr)_2_POCH(NMe_2_)OEt] with diethyl phosphite in 74% yield [35].

In the present paper we report a novel method for α-phosphonylation, α-phosphinylation or α-phosphinoylation of 1-(*N*-acetylamino)alkylphosphonates that enables effective transformation of the starting compounds into 1-aminoalkylidenebisphosphonates **1**, 1-amino-1-phosphinylalkylphosphonates **3**, or 1-amino-1-phosphinoylalkylphosphonates **4**, respectively. The two latter groups of compounds are not available by common methods used for the synthesis of 1-aminobisphosphonic acid derivatives.

## Results and Discussion

**Scheme 1:**
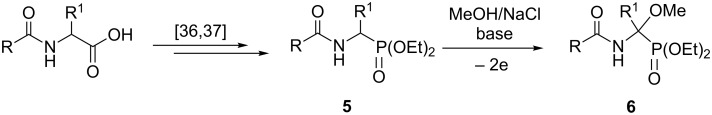
Electrochemical α-methoxylation of 1-(*N*-acylamino)alkylphosphonates.

Recently we developed a simple and effective two-step transformation of *N*-acyl-α-amino acids into their phosphonic analogues that allows for easy access to a variety of structurally diversified *N*-acyl-α-aminophosphonates **5** (Scheme 1) [36–37].

Despite the electron-withdrawing inductive effect of *N*-acylamino- and dialkoxyphosphonyl groups of 1-aminophosphonates **5**, the electrophilicity of these compounds’ α-carbon is displayed only after functionalisation of the α-position with a nucleofuge group, e.g. by α-bromination of 1-aminophosphonates with *N*-bromosuccinimide [28,38–41] or by alkoxylation or aryloxylation of this position [20,29–30].

As we demonstrated, electrophilic activation of the α-carbon of 1-aminophosphonates can easily be achieved by electrochemical α-methoxylation of these compounds in methanol, mediated with NaCl (Scheme 1, Table 2). α-Methoxylations were carried out in the presence of sodium methoxide or 3-(1-piperidino)propyl-functionalised silica gel (SiO_2_-Pip) or without any base. The composition of the reaction mixture was controlled using ^1^H NMR to determine the optimal electric charge consumption. The best result of α-methoxylation of diethyl *N*-acetylaminomethylphosphonate (**5a**) was achieved without using any base, whereas the best result of the same reaction of the analogous ethylphosphonic acid derivative **5b** was obtained in the presence of sodium methoxide. Attempts to carry out the analogous α-methoxylation for phosphonic derivatives of valine and phenylalanine failed, probably due to the steric hindrance exerted by a bulky substituent at the α-position.

**Table 2:** Electrochemical α-methoxylation of diethyl 1-(*N*-acetylamino)alkylphosphonates **5** (R = Me).

substrate **5**		procedure	base	charge [F/mol]	product **6**	
				
no.	R^1^				no.	yield^a^ [%]

**5a**	H	A	–	4.5	**6a**	88
**5a**	H	B	MeONa	4.5	**6a**	41
**5b**	Me	A	–	10	**6b**	66
**5b**	Me	B	SiO_2_-Pip	10	**6b**	63
**5b**	Me	B	MeONa	10	**6b**	72

^a^The yield was estimated based on the ^1^H NMR spectrum of the reaction mixture relative to dimethyldiphenylsilane as the internal standard.

Electrochemical α-alkoxylation of *N*-acyl-α-amino acid esters mediated by NaCl, NaBr, LiCl, KCl or KI is a well-known reaction [38,42–46], whereas the analogous reaction of *N*-acyl-α-aminophosphonic acid esters was mentioned in the literature only once, in relation to α-methoxylation of diethyl *N*-benzoylaminomethylphosphonate [42].

**Scheme 2:**
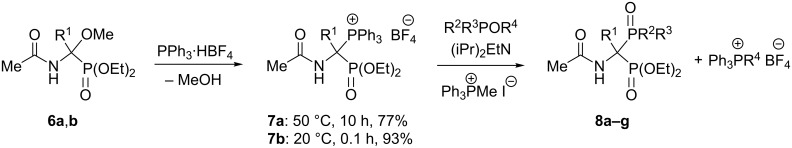
Transformation of diethyl 1-(*N*-acetylamino)-1-methoxyalkylphosphonates into bisphosphoric acid esters via the corresponding phosphonium salts.

Attempts to carry out a Michaelis–Arbuzov-like reaction of the obtained diethyl 1-(*N*-acetylamino)-1-methoxyalkylphosphonates with triethyl phosphite failed due to still too low electrophilic activity of the α-carbon atom of the removed 1-methoxyalkylphosphonic acid derivatives. However, the electrophilic activity of the latter compounds was enough for their reaction with triphenylphosphine. Therefore, to enhance the electrophilic activity of the discussed 1-methoxyalkylphosphonic acid derivatives, their methoxy group was successfully displaced by the triphenylphosphonium group. Thus, heating the homogeneous mixture of diethyl 1-(*N*-acetylamino)-1-methoxymethylphosphonate (**6a**) with triphenylphosphonium tetrafluoroborate at 50 °C gave hitherto unknown phosphonium salt **7a** as a resin-like compound stable at room temperature (Scheme 2). In the case of 1-(*N*-acetylamino)-1-methoxyethylphosphonate (**6b**), the analogous reaction with triphenylphosphonium tetrafluoroborate proceeded very quickly, without heating, but the resulting phosphonium salt **7b** underwent slow decomposition even at low temperature (Scheme 2). The most characteristic feature of the ^13^C NMR spectra of these compounds was a doublet of doublets in the range of 45–58 ppm (*J*_1_ = 42–49 Hz, *J*_2_* =* 151–160 Hz) assigned to the α-carbon atom coupled with two nonequivalent phosphorus atoms as well as the presence of two doublets of phosphorus atoms in the ranges of 11.7–16.5 and 28.5–37.0 ppm (*J =* 33–48 Hz) in the ^31^P NMR spectra.

Finally, it was demonstrated that diethyl 1-(*N*-acetylamino)-1-triphenylphosphoniumalkylphosphonate tetrafluoroborates **7** react smoothly with trialkyl phosphites, dialkyl phosphonites or alkyl phosphinites in the presence of Hünig’s base and methyltriphenylphosphonium iodide as catalysts to give bisphosphonates **8a,b**, 1-phosphinylalkylphosphonates **8c–e** or 1-phosphinoylalkylphosphonates **8f,g**, respectively, in good yields (Table 3). The nature of the catalytic activity of Hünig’s base and methyltriphenylphosphonium iodide in similar Michaelis–Arbuzov-like reactions of 1-(*N*-acylamino)alkylphosphonium salts was explained in our previous paper [47].

**Table 3:** Reaction of diethyl 1-(*N*-acetylamino)-1-triphenylphosphoniumalkylphosphonate tetrafluoroborates **7** with phosphorus nucleophiles.

substrate			nucleophile			temp. [°C]	time [h]	product	
				
no.	R^1^		R^2^	R^3^	R^4^			no.	yield [%]

**7a**	H		EtO	EtO	Et	60	5	**8a**	75
**7b**	Me		EtO	EtO	Et	20	2.5	**8b**	70
**7a**	H		EtO	Me	Et	20	22	**8c**^a^	50^b^
**7a**	H		EtO	Ph	Et	60	6	**8d**	51^c^
**7b**	Me		EtO	Ph	Et	20	3	**8e**	53^c^
**7a**	H		Ph	Ph	Me	60	2	**8f**	68
**7b**	Me		Ph	Ph	Me	20	2	**8g**	76

^a^Synthesis was performed under argon due to the sensitivity of the nucleophile to oxidation. ^b^Mixture of diastereomers in a ratio of 1:1. ^c^Mixture of diastereomers in a ratio of 1.4:1.

## Conclusion

It was demonstrated that effective electrophilic activation of the α-position of 1-(*N*-acetylamino)alkylphosphonates can be achieved by electrochemical α-methoxylation of these compounds in methanol, mediated with NaCl, followed by displacement of the methoxy group with triphenylphosphonium tetrafluoroborate to give hitherto unknown 1-(*N*-acetylamino)-1-triphenylphosphoniumalkylphosphonate tetrafluoroborates. The Michaelis–Arbuzov-like reaction of the latter compounds with trialkyl phosphites, dialkyl phosphonites or alkyl phosphinites in the presence of Hünig’s base and methyltriphenylphosphonium iodide gave the expected alkylidenebisphosphonates, 1-phosphinylalkylphosphonates or 1-phosphinoylalkylphosphonates, respectively, in good yields. The reported set of reactions enables α-phosphonylation, α-phosphinylation or α-phosphinoylation of 1-(*N*-acylamino)alkylphosphonates.

## Experimental

**General methods**: IR spectra were measured on a FTIR spectrophotometer (ATR method). ^1^H and ^13^C NMR spectra were recorded at operating frequencies of 400 or 600 MHz and 100 MHz, respectively, using TMS as the resonance shift standard. ^31^P NMR spectra were recorded at an operating frequency of 162 MHz, with 80% orthophosphoric acid as an external resonance shift standard. All chemical shifts (δ) are reported in ppm, and coupling constants (*J*) are in Hz. Spectroscopic properties of all synthesized compounds as well as ^1^H NMR, ^13^C NMR and ^31^P NMR spectra of selected bisphosphoric acid esters are given in Supporting Information File 1.

**Electrochemical α-methoxylation of diethyl 1-(*N*-acetylamino)alkylphosphonates 5. Procedure A:** MeOH (10 mL) and diethyl 1-(*N*-acetylamino)alkylphosphonate **5** (0.3 mmol) were added to a glass beaker (25 mL) equipped with a cylindrical Pt mesh anode (20 cm^2^) and a rotating flat cathode (platinised titanium, 1.2 cm^2^). In the case of 1-(*N*-acetylamino)methylphosphonate (**5a**), NaCl (70 mg, 1.2 mmol) was placed in the beaker in its entirety at the beginning of oxidation, as opposed to 1-(*N*-acetylamino)ethylphosphonate (**5b**), where the mediator was added in three portions during the process (0.6 mmol at the start and 2 × 0.3 mmol – each portion after the charge of 3.0 F/mol had been passed). The beaker was placed in an ice-water bath and its content was stirred for 5 min by means of a rotating cathode before commencement of the oxidation. Electrolysis was carried out while stirring at 0.1 A until a 4.5–10 F/mol charge had passed (Table 2). After evaporation of the methanol in vacuo, the residue was extracted with CH_2_Cl_2_. Subsequent evaporation of the solvent from the extracts under reduced pressure yielded 1-(*N*-acetylamino)-1-methoxyalkylphosphonates **6**, which were used in the next step without further purification.

**Procedure B:** MeOH (10 mL), diethyl 1-(*N*-acetylamino)alkylphosphonate **5** (0.3 mmol), and NaCl (70 mg, 1.2 mmol), in one portion for 1-(*N*-acetylamino)methylphosphonate (**5a**) or in three portions for derivative **5b** (as described above for Procedure A), and SiO_2_-Pip (150 mg, 0.15 mmol) or MeONa (0.6 mL methanolic solution, 0.3 mmol), were added to a glass beaker (25 mL) equipped with a cylindrical Pt mesh anode (20 cm^2^) and a rotating flat cathode (platinised titanium, 1.2 cm^2^). Electrolysis was carried out while stirring at 0.1 A until a 4.5–10 F/mol charge had passed (Table 2). After evaporation of MeOH under reduced pressure, the residue was extracted with CH_2_Cl_2_ followed by further evaporation of the solvent from the extracts in vacuo to obtain diethyl 1-(*N*-acetylamino)-1-methoxyalkylphosphonates **6**.

**Synthesis of 1-(*N*-acetylamino)-1-triphenylphosphoniummethylphosphonate tetrafluoroborate 7a.** Triphenylphosphonium tetrafluoroborate (52.5 mg, 0.15 mmol) was added to a solution of diethyl 1-(*N*-acetylamino)-1-methoxymethylphosphonate (**6a**, 36 mg, 0.15 mmol) in CH_2_Cl_2_ (1 mL). After obtaining a homogeneous solution, the solvent was evaporated to dryness and the residue was heated in a flask equipped with a septum with a needle for 20 h at 50 °C to obtain compound **7a**, which was used in the subsequent synthesis without further purification.

**Synthesis of 1-(*N*-acetylamino)-1-triphenylphosphoniumethylphosphonate tetrafluoroborate 7b.** Triphenylphosphonium tetrafluoroborate (52.5 mg, 0.15 mmol) was added to a solution of diethyl 1-(*N*-acetylamino)-1-methoxyethylphosphonate (**6b**, 38 mg, 0.15 mmol) in dichloromethane (1 mL). The homogeneous mixture was stirred at room temperature for 0.1 h, and then the solvent was evaporated to dryness to give product **7b**, which was used in the subsequent synthesis without further purification.

**Synthesis of bisphosphonates 8a,b, 1-phosphinylalkylphosphonates 8c–e and 1-phosphinoylalkylphosphonates 8f,g. General procedure:** Methyltriphenylphosphonium iodide (36 mg, 0.088 mmol), phosphorus nucleophile (0.52 mmol) and (iPr)_2_EtN (6.0 μL, 0.035 mmol) were added to a solution of 1-(*N*-acetylamino)-1-triphenylphosphoniumalkylphosphonate tetrafluoroborate **7** (0.35 mmol) in CH_2_Cl_2_ (1 mL) in a glass vial with a screw-cap. For syntheses started from 1-(*N*-acetylamino)-1-triphenylphosphoniummethylphosphonate tetrafluoroborate (**7a**), the mixture was heated at 60 °C for the time shown in Table 3. For the syntheses using 1-(*N*-acetylamino)-1-triphenylphosphoniumethylphosphonate tetrafluoroborate (**7b**), the mixture was left at room temperature for 2–3 h (Table 3). The solvent was evaporated under reduced pressure, the residue was extracted with toluene, and the toluene was subsequently evaporated. The crude products were purified by column chromatography (silica gel, CH_2_Cl_2_/MeOH for **8a–f**, and AcOEt/MeOH for **8g**).

## Supporting Information

File 1 Experimental and analytical data.
