# Supplementary material for: A new method for the synthesis of α-aminoalkylidenebisphosphonates and their asymmetric phosphonyl-phosphinyl and phosphonyl-phosphinoyl analogues
Source: Beilstein J Org Chem. 2015 Aug 13;11:1418–24. doi: 10.3762/bjoc.11.153 (PMC4578414; doi:10.3762/bjoc.11.153)
Supplement: File 1 — Experimental and analytical data. [file Beilstein_J_Org_Chem-11-1418-s001.pdf]

**Supporting Information**

**for**

# A new method for the synthesis of α-aminoalkylidene-bisphosphonates and their asymmetric phosphonyl-phosphinyl and phosphonyl-phosphinoyl analogues

Anna Kuźnik,[1*§] Roman Mazurkiewicz,[1] Mirosława Grymel,[1] Katarzyna Zielińska,[1] Jakub Adamek,[1] Ewa Chmielewska,[2] Marta Bochno,[2] Sonia Kubica[1]

Address: [1]Department of Organic Chemistry, Biochemistry and Biotechnology, Silesian University of Technology, B. Krzywoustego 4, 44-100 Gliwice, Poland and [2]Department of Bioorganic Chemistry, Wrocław University of Technology, Wybrzeże Wyspiańskiego 27, 50-370 Wrocław, Poland

Email: Anna Kuźnik - Anna.Kuznik@polsl.pl

[*]Corresponding author

[§]Tel: +48 32 237 16 13; Fax: +48 32 237 20 94.

## Experimental and analytical data

### Table of contents

1. Spectroscopic properties of all synthesized compounds **6–8**……………………… S2–S5
2. $^{1}$H NMR, $^{13}$C NMR and $^{31}$P NMR spectra of selected bisphosphoric acid esters **8**.. S6–S17

**Diethyl 1-(*N*-acetylamino)-1-methoxymethylphosphonate (6a).** Pale yellow oil. $^{1}$H NMR (400 MHz, $CDCl_3$) $\delta$ 6.63 (br d, $J$ = 6.8 Hz, 1H), 5.44 (dd, $J_1$ = 8.4, $J_2$ = 10.4 Hz, 1H), 4.25-4.12 (m, 4H)[a], 3.43 (s, 3H), 2.10 (s, 3H), 1.35 (t, $J$ = 7.2 Hz, 3H)[b], 1.32 (t, $J$ = 7.0 Hz, 3H)[b] ppm; $^{13}$C NMR (100 MHz, $CDCl_3$) $\delta$ 170.8, 76.1 (d, $J$ = 199.6 Hz), 63.8 (d, $J$ = 6.7 Hz)[c] and 63.1 (d, $J$ = 6.8 Hz)[c], 57.4 (d, $J$ = 14.4 Hz), 23.2, 16.44 (d, $J$ = 5.3 Hz)[b] and 16.39 (d, $J$ = 5.3 Hz)[b] ppm; $^{31}$P NMR (162 MHz, $CDCl_3$) $\delta$ 16.8 ppm; IR (ATR) 3257, 1690, 1235, 1020 cm$^{-1}$. HRMS (TOF-ESI) calcd for $C_8H_{18}NO_5PNa$ $[M + Na]^+$ 262.0820, found 262.0825. [a]Overlapping signals of two diastereotopic Et groups ($O\underline{CH_2}CH_3$). [b]Diastereotopic Et group ($OCH_2\underline{CH_3}$). [c]Diastereotopic Et group ($O\underline{CH_2}CH_3$).

**Diethyl 1-(*N*-acetylamino)-1-methoxyethylphosphonate (6b).** Pale yellow oil. $^{1}$H NMR (400 MHz, $CDCl_3$) $\delta$ 6.14 (br s, 1H), 4.29-4.06 (m, 4H)[a], 3.38 (s, 3H), 2.05 (s, 3H), 1.93 (d, $J$ = 15.2 Hz, 3H), 1.36 (t, $J$ = 7.2 Hz, 3H)[b], 1.35 (t, $J$ = 7.0 Hz, 3H)[b] ppm; $^{13}$C NMR (100 MHz, $CDCl_3$) $\delta$ 170.6, 85.0 (d, $J$ = 195.0 Hz), 63.9 (d, $J$ = 6.9 Hz)[c] and 63.1 (d, $J$ = 6.9 Hz)[c], 50.4 (d, $J$ = 9.8 Hz), 24.8, 18.2, 16.32 (d, $J$ = 5.3 Hz)[b] and 16.30 (d, $J$ = 6.1 Hz)[b] ppm; $^{31}$P NMR (162 MHz, $CDCl_3$) $\delta$ 19.3 ppm; IR (ATR) 3277, 1679, 1239, 1162, 1018 cm$^{-1}$. HRMS (TOF-ESI) calcd for $C_9H_{20}NO_5PNa$ $[M + Na]^+$ 276.0977, found 276.0973. [a]Overlapping signals of two diastereotopic Et groups ($O\underline{CH_2}CH_3$). [b]Diastereotopic Et group ($OCH_2\underline{CH_3}$). [c]Diastereotopic Et group ($O\underline{CH_2}CH_3$).

**Diethyl 1-(*N*-acetylamino)-1-triphenylphosphoniummethylphosphonate tetrafluoroborate (7a).** Pale yellow resin. $^{1}$H NMR (400 MHz, $CDCl_3$) $\delta$ 8.08 (br d, $J$ = 9.2 Hz, 1H), 7.87-7.49 (m, 15H), 6.41 (ddd, $J_1$ = 9.6, $J_2$ = 16.0, $J_3$ = 21.6 Hz, 1H), 4.33-4.07 (m, 4H)[a], 1.91 (s, 3H), 1.31 (t, $J$ = 7.0 Hz, 3H)[b], 1.15 (t, $J$ = 7.0 Hz, 3H)[b], ppm; $^{13}$C NMR (100 MHz, $CDCl_3$) $\delta$ 171.6 (d, $J$ = 2.3 Hz), 135.3 (d, $J$ = 3.1 Hz), 133.0 (d, $J$ = 10.5 Hz), 130.1 (d, $J$ = 13.0 Hz), 116.5 (d, $J$ = 85.1 Hz), 65.0 (d, $J$ = 7.8 Hz)[c] and 64.9 (d, $J$ = 6.7 Hz)[c], 44.9 (dd, $J_1$ = 49.3, $J_2$ = 151.0 Hz), 21.9, 16.2 (d, $J$ = 5.3 Hz)[b] and 15.9 (d, $J$ = 4.6 Hz)[b] ppm; $^{31}$P NMR (162 MHz, $CDCl_3$) $\delta$ 28.5 (d, $J$ = 34.5 Hz), 11.7 ($J$ = 33.2 Hz) ppm; IR (ATR) 3359, 1667, 1270, 1015 cm$^{-1}$. HRMS (TOF-ESI) calcd for $C_{25}H_{30}NO_4P_2$ $[M]^+$ 470.1650, found 470.1647. [a]Overlapping signals of two diastereotopic Et groups ($O\underline{CH_2}CH_3$). [b]Diastereotopic Et group ($OCH_2\underline{CH_3}$). [c]Diastereotopic Et group ($O\underline{CH_2}CH_3$).

**Diethyl 1-(*N*-acetylamino)-1-triphenylphosphoniumethylphosphonate tetrafluoroborate (7b).** Pale yellow resin. $^{1}$H NMR (400 MHz, $CDCl_3$) $\delta$ 8.15-8.12 (br dd, $J_1$ = 3.6, $J_2$ = 8.0 Hz, 1H), 7.92-7.49 (m, 15H), 4.23-4.09 (m, 4H)[a], 1.93 (dd, $J_1$ = 15.8, $J_2$ = 19.0 Hz, 3H), 1.72 (s, 3H), 1.40-1.26 (m, 6H)[b] ppm; $^{13}$C NMR (100 MHz, $CDCl_3$) $\delta$ 171.2, 135.2 (d, $J$ = 10.0 Hz), 134.3 (d, $J$ = 3.0 Hz), 129.4 (d, $J$ = 13.0 Hz), 120.3 (d, $J$ = 84.2 Hz), 65.2 (d, $J$ = 7.6 Hz)[c] and 65.1 (d, $J$ = 8.3 Hz)[c], 58.2 (dd, $J_1$ = 42.4, $J_2$ = 160.1 Hz), 21.6, 16.4, 16.4 (d, $J$ = 5.3 Hz)[d] and 16.2 (d, $J$ = 5.3 Hz)[d] ppm; $^{31}$P NMR (162 MHz, $CDCl_3$) $\delta$ 37.0 (d, $J$ = 48.1 Hz), 16.5 (d, $J$ = 48.1 Hz) ppm; IR (ATR) 3325, 1679, 1248, 1163, 1017 cm$^{-1}$. HRMS (TOF-ESI) calcd for $C_{26}H_{32}NO_4P_2$ $[M]^+$ 484.1807, found 484.1807. [a]Overlapping signals of two diastereotopic Et groups ($O\underline{CH_2}CH_3$). [b]Overlapping signals of two diastereotopic Et groups ($OCH_2\underline{CH_3}$). [c]Diastereotopic Et group ($O\underline{CH_2}CH_3$). [d]Diastereotopic Et group ($OCH_2\underline{CH_3}$).

**Tetraethyl 1-(*N*-acetylamino)methylidenebisphosphonate (8a).** White crystals, mp 25°C. $^{1}$H NMR (400 MHz, $CDCl_3$) $\delta$ 6.30 (br d, $J$ = 10.4 Hz, 1H), 5.04 (ddd, $J_1$ = 10.4, $J_2$ = 21.6, $J_3$ = 21.9 Hz, 1H), 4.25-4.14 (m, 8H)[a], 2.08 (d, $J$ = 0.9 Hz, 3H), 1.34 (t, $J$ = 7.0 Hz, 6H)[b], 1.33 (t, $J$ = 7.0 Hz, 6H)[b] ppm; $^{13}$C NMR (100 MHz, $CDCl_3$) $\delta$ 169.0 (t, $J$ = 4.2 Hz), 63.67 (d, $J$ = 3.1 Hz)[c] and 63.64 (d, $J$ = 3.0 Hz)[c] and 63.49 (d, $J$ = 3.8 Hz)[c] and 63.46 (d, $J$ = 3.0 Hz)[c], 43.6 (t, $J$ = 146.5 Hz), 22.8, 16.36 (d, $J$ = 3.0 Hz)[d] and 16.33 (d, $J$ = 3.1 Hz)[d] and 16.30

(d, $J$ = 3.0 Hz)[d], 16.27 (d, $J$ = 3.0 Hz)[d] ppm; $^{31}$P NMR (162 MHz, $CDCl_3$) $\delta$ 17.0 ppm; IR (ATR) 3252, 1663, 1242, 1014 $cm^{-1}$. HRMS (TOF-ESI) calcd for $C_{11}H_{25}NO_7P_2Na$ $[M + Na]^+$ 368.1004, found 368.1001. [a]Overlapping signals of four diastereotopic Et groups ($O\underline{CH_2}CH_3$). [b]Two diastereotopic Et groups ($OCH_2\underline{CH_3}$). [c]Diastereotopic Et group ($O\underline{CH_2}CH_3$). [d]Diastereotopic Et group ($OCH_2\underline{CH_3}$).

**Tetraethyl 1-(*N*-acetylamino)ethylidenebisphosphonate (8b).** Colourless oil. $^1$H NMR (400 MHz, $CDCl_3$) $\delta$ 6.02 (br t, $J$ = 5.0 Hz, 1H), 4.28-4.18 (m, 8H)[a], 2.01 (s, 3H), 1.98 (t, $J$ = 16.8 Hz, 3H), 1.36 (t, $J$ = 7.2 Hz, 12H) ppm; $^{13}$C NMR (100 MHz, $CDCl_3$) $\delta$ 169.5 (t, $J$ = 5.3 Hz), 63.76 (d, $J$ = 3.1 Hz)[b] and 63.73 (d, $J$ = 3.7 Hz)[b], 56.8 (t, $J$ = 146.0 Hz), 24.6, 17.0 (t, $J$ = 4.5 Hz), 16.42 (d, $J$ = 2.3 Hz)[c] and 16.39 (d, $J$ = 3.0 Hz)[c] and 16.36 (d, $J$ = 2.3 Hz)[c] and 16.34 (d, $J$ = 3.0 Hz)[c] ppm; $^{31}$P NMR (162 MHz, $CDCl_3$) $\delta$ 20.5 ppm; IR (ATR) 3273, 1690, 1238, 1019 $cm^{-1}$. HRMS (TOF-ESI) calcd for $C_{12}H_{27}NO_7P_2Na$ $[M + Na]^+$ 382.1160, found 382.1163. [a]Overlapping signals of four diastereotopic Et groups ($O\underline{CH_2}CH_3$). [b]Diastereotopic Et groups ($O\underline{CH_2}CH_3$). [c]Diastereotopic Et group ($OCH_2\underline{CH_3}$).

**Diethyl 1-(*N*-acetylamino)-1-[ethoxy(methylphosphinyl)]methylphosphonate (8c)[a].** Colourless oil. $^1$H NMR (600 MHz, $CDCl_3$) $\delta$ 6.84 (br s, 1H)[b], 6.50 (br d, $J$ = 9.6 Hz, 1H)[b], 4.97 (ddd, $J_1$ = 10.2, $J_2$ = 16.2, $J_3$ = 26.4 Hz, 1H)[b], 4.95 (ddd, $J_1$ = 10.2, $J_2$ = 14.4, $J_3$ = 24.6 Hz, 1H)[b], 4.26-4.05 (m, 6H)[c], 2.10 (s, 3H)[b] and 2.09 (s, 3H)[b], 1.71 (d, $J$ = 14.4 Hz, 3H)[b], 1.59 (d, $J$ = 14.4 Hz, 3H)[b], 1.36-1.29 (m, 9H)[d] ppm; $^{13}$C NMR (100 MHz, $CDCl_3$) $\delta$ 169.5 (dd, $J_1$ = 3.0, $J_2$ = 4.5 Hz)[b], 169.2 (dd, $J_1$ = 3.1, $J_2$ = 3.8 Hz)[b], (63.71, 63.65, 63.62, 63.55, 63.48, 62.15, 62.08, 61.95, 61.88)[e], 46.5 (dd, $J_1$ = 86.1, $J_2$ = 143.4 Hz)[b], 46.0 (dd, $J_1$ = 85.2, $J_2$ = 143.5 Hz)[b], 22.87[b], 22.79[b], (16.63, 16.58, 16.49, 16.43, 16.38, 16.37, 16.32, 16.28, 16.26, 16.22)[f], 14.6 (d, $J$ = 97.1 Hz)[b], 13.6 (dd, $J_1$ = 3.1, $J_2$ = 100.1 Hz)[b] ppm; $^{31}$P NMR (162 MHz, $CDCl_3$) $\delta$ 46.1 (d, $J$ = 20.9 Hz), 45.2 (d, $J$ = 30.8 Hz), 17.5 (d, $J$ = 21.1 Hz), 17.4 (d, $J$ = 30.8 Hz) ppm; IR (ATR) 3200, 1672, 1215, 1023 $cm^{-1}$. HRMS (TOF-ESI) calcd for $C_{10}H_{23}NO_6P_2Na$ $[M + Na]^+$ 338.0898, found 338.0917. [a]The mixture of two diastereomers in the ratio of 1:1. [b]Separate signals of one diastereomer. [c]Overlapping signals of two diastereotopic and one heterotopic Et groups ($O\underline{CH_2}CH_3$). [d]Overlapping signals of two diastereotopic and one heterotopic Et groups ($OCH_2\underline{CH_3}$). [e]Partly overlapping doublets of two diastereotopic and one heterotopic Et groups of two diastereomers ($O\underline{CH_2}CH_3$). [f]Partly overlapping signals of two diastereotopic and one heterotopic Et groups of two diastereomers ($OCH_2\underline{CH_3}$).

**Diethyl 1-(*N*-acetylamino)-1-[ethoxy(phenylphosphinyl)]methylphosphonate (8d)[a].** Colourless oil. $^1$H NMR (600 MHz, $CDCl_3$) $\delta$ 7.87-7.81 (m, 2H)[b], 7.60-7.56 (m, 1H)[b], 7.51-7.46 (m, 2H)[b], 6.32 (br d, $J$ = 9.6 Hz, 1H)[c,d], 6.13 (br d, $J$ = 10.2 Hz, 1H)[c,e], 5.144 (ddd, $J_1$ = 10.2, $J_2$ = 18.0, $J_3$ = 36.6 Hz, 1H)[c,e], 5.143 (ddd, $J_1$ = 5.7, $J_2$ = 10.8, $J_3$ = 17.4 Hz, 1H)[c,d], 4.28-4.01 (m, 6H)[b,f], 2.02 (s, 3H)[c,d] and 1.81 (s, 3H)[c,e], 1.37-1.31 (m, 6H)[b,g], 1.24 (t, $J$ = 7.1 Hz, 3H)[c,d,h], 1.22 (t, $J$ = 7.0 Hz, 3H)[c,e,h] ppm; $^{13}$C NMR (100 MHz, $CDCl_3$) $\delta$ 168.9 (dd, $J_1$ = 3.8, $J_2$ = 3.8 Hz)[c,d], 168.6 (dd, $J_1$ = 3.8, $J_2$ = 3.8 Hz)[c,e], [133.0 (d, $J$ = 3.0 Hz), 132.4 (d, $J$ = 9.8 Hz), 128.9 (dd, $J_1$ = 1.5, $J_2$ = 132.8 Hz), 128.44 (d, $J$ = 16.0 Hz)][d,i] and [132.9 (d, $J$ = 2.3 Hz), 132.2 (d, $J$ = 9.8 Hz), 128.7 (dd, $J_1$ = 4.9, $J_2$ = 134.7 Hz), 128.44 (d, $J$ = 11.4 Hz)][e,i], (63.4, 63.35, 63.3, 63.2, 62.6, 62.5, 62.1, 62.0)[j], 46.9 (dd, $J_1$ = 95.6, $J_2$ = 146.5 Hz)[c,d], 46.0 (dd, $J_1$ = 100.9, $J_2$ = 143.4 Hz)[c,e], 22.84[c,d], 22.54[c,e], (16.54, 16.48, 16.46, 16.40, 16.35, 16.30, 16.25, 16.21, 16.15)[k] ppm; $^{31}$P NMR (162 MHz, $CDCl_3$) $\delta$ 34.8 (d, $J$ = 16.0 Hz), 33.6 (d, $J$ = 28.4 Hz), 17.4 (d, $J$ = 16.0 Hz), 16.9 (d, $J$ = 29.5 Hz) ppm; IR (ATR) 3447, 2984, 1667, 1225, 1022 $cm^{-1}$. HRMS (TOF-ESI) calcd for $C_{15}H_{25}NO_6P_2Na$ $[M + Na]^+$ 400.1055, found 400.1059. [a]The mixture of two diastereomers in the ratio of 1:1.4. [b]Overlapping signals of two diastereomers. [c]Separate signal of one diastereomer. [d]Minor

diastereomer. [e]Major diastereomer. [f]Overlapping signals of two diastereotopic and one heterotopic Et groups ($O\underline{CH_2}CH_3$). [g]Overlapping signals of two diastereotopic Et groups ($OCH_2\underline{CH_3}$). [h]Heterotopic Et group ($OCH_2\underline{CH_3}$). [i]Separate signal of PhP=O group of one diastereomer. [j]Partly overlapping doublets of two diastereotopic and one heterotopic Et groups of two diastereomers ($O\underline{CH_2}CH_3$). [k]Partly overlapping signals of two diastereotopic and one heterotopic Et groups of two diastereomers ($OCH_2\underline{CH_3}$).

**Diethyl 1-(*N*-acetylamino)-1-[ethoxy(phenylphosphinyl)]ethylphosphonate (8e).** Major diastereomer. Colourless oil. $^{1}$H NMR (400 MHz, $CDCl_3$) $\delta$ 7.87-7.81 (m, 2H), 7.60-7.46 (m, 3H), 6.19 (br s, 1H), 4.31-4.10 (m, 6H)[a], 1.91 (s, 3H), 1.89 (dd, $J_1$ = 16.4, $J_2$ = 16.8 Hz, 3H), 1.40-1.30 (m, 9H)[b] ppm; $^{13}$C NMR (100 MHz, $CDCl_3$) $\delta$ 169.6 (dd, $J_1$ = 4.5, $J_2$ = 5.3 Hz), 133.3 (d, $J$ = 9.1 Hz), 132.9 (d, $J$ = 3.1 Hz), 128.2 (d, $J$ = 12.1 Hz), 128.0 (dd, $J_1$ = 3.4, $J_2$ = 131.7 Hz), [63.7 (d, $J$ = 7.6 Hz), 63.5 (d, $J$ = 7.6 Hz) and 62.7 (d, $J$ = 6.8 Hz)][c], 58.5 (dd, $J_1$ = 97.6, $J_2$ = 144.6 Hz), 24.6, (17.15, 17.13, 17.10, 17.09)[d], 16.45 (d, $J$ = 6.0 Hz)[e] and 16.37 (d, $J$ = 5.3 Hz)[e] ppm; $^{31}$P NMR (162 MHz, $CDCl_3$) $\delta$ 39.4 (d, $J$ = 16.0 Hz), 20.8 (d, $J$ = 15.9 Hz) ppm; IR (ATR) 3277, 1558, 1184, 1023 cm$^{-1}$. HRMS (TOF-ESI)[f] calcd for $C_{16}H_{27}NO_6P_2Na$ $[M + Na]^+$ 414.1211, found 414.1208. [a]Overlapping signals of two diastereotopic and one heterotopic Et groups ($O\underline{CH_2}CH_3$). [b]Overlapping signals of two diastereotopic and one heterotopic Et groups ($OCH_2\underline{CH_3}$). [c]Signals of two diastereotopic and one heterotopic Et groups ($O\underline{CH_2}CH_3$). [d]Overlapping signals of the Me group at $C_\alpha$ position and heterotopic Et group ($OCH_2\underline{CH_3}$). [e]Diastereotopic Et group ($OCH_2\underline{CH_3}$). [f]MS spectrum was performed for the mixture of diastereomers.

**Diethyl 1-(*N*-acetylamino)-1-[ethoxy(phenylphosphinyl)]ethylphosphonate (8e).** Minor diastereomer. Colourless oil. $^{1}$H NMR (400 MHz, $CDCl_3$) $\delta$ 7.88-7.83 (m, 2H), 7.62-7.48 (m, 3H), 6.36 (br s, 1H), 4.29-4.05 (m, 6H)[a], 2.02 (s, 3H), 1.93 (dd, $J_1$ = 16.8, $J_2$ = 17.2 Hz, 3H), [1.37 (t, $J$ = 7.0 Hz, 3H), 1.30 (t, $J$ = 7.0 Hz, 3H) and 1.23 (t, $J$ = 7.0 Hz, 3H)][b] ppm; $^{13}$C NMR (100 MHz, $CDCl_3$) $\delta$ 170.0, 133.5 (d, $J$ = 9.1 Hz), 133.0 (d, $J$ = 2.2 Hz), 128.2 (d, $J$ = 12.9 Hz), 127.7 (d, $J$ = 131.3 Hz), [63.8 (d, $J$ = 7.5 Hz), 63.5 (d, $J$ = 7.6 Hz) and 62.6 (d, $J$ = 6.8 Hz)][c], 58.7 (dd, $J_1$ = 94.1, $J_2$ = 146.4 Hz), 24.8, 16.9 (d, $J$ = 3.2 Hz), [16.49 (d, $J$ = 6.0 Hz), 16.43 (d, $J$ = 6.1 Hz) and 16.22 (d, $J$ = 6.1 Hz)][b] ppm; $^{31}$P NMR (162 MHz, $CDCl_3$) $\delta$ 38.2 (d, $J$ = 27.5 Hz), 20.1 (d, $J$ = 27.5 Hz) ppm; IR (ATR) 3268, 1689, 1227, 1019 cm$^{-1}$. [a]Overlapping signals of two diastereotopic and one heterotopic Et groups ($O\underline{CH_2}CH_3$). [b]Signals of two diastereotopic and one heterotopic Et groups ($OCH_2\underline{CH_3}$). [c]Signals of two diastereotopic and one heterotopic Et groups ($O\underline{CH_2}CH_3$).

**Diethyl 1-(*N*-acetylamino)-1-(diphenylphosphinoyl)methylphosphonate (8f).** White resin. $^{1}$H NMR (600 MHz, $CDCl_3$) $\delta$ 7.92-7.88 (m, 2H), 7.84-7.81 (m, 2H), 7.55-7.43 (m, 6H), 6.71 (br d, $J$ = 9.6 Hz, 1H), 5.62 (ddd, $J_1$ = 9.6, $J_2$ = 10.2, $J_3$ = 21.6 Hz, 1H), 4.13-3.93 (m, 4H)[a], 1.85 (s, 3H), 1.23 (dt, $J_1$ = 0.6, $J_2$ = 7.5 Hz, 3H)[b] and 1.12 (t, $J$ = 7.2 Hz, 3H)[b] ppm; $^{13}$C NMR (100 MHz, $CDCl_3$) $\delta$ 169.4 (dd, $J_1$ = 3.1, $J_2$ = 3.8 Hz), [132.2 (d, $J$ = 3.0 Hz), 132.1 (d, $J$ = 3.0 Hz), 131.4 (d, $J$ = 9.8 Hz), 131.0 (d, $J$ = 9.9 Hz), 130.71 (dd, $J_1$ = 7.6, $J_2$ = 133.9 Hz), 130.68 (d, $J$ = 100.2 Hz), 128.5 (d, $J$ = 12.2 Hz) and 128.4 (d, $J$ = 12.2 Hz)][c], 63.3 (d, $J$ = 6.8 Hz)[d], 62.9 (d, $J$ = 6.1 Hz)[d], 46.4 (dd, $J_1$ = 66.4, $J_2$ = 146.1 Hz), 22.4, 16.2 (d, $J$ = 6.0 Hz)[b] and 15.9 (d, $J$ = 6.1 Hz)[b] ppm; $^{31}$P NMR (162 MHz, $CDCl_3$) $\delta$ 30.6 (d, $J$ = 16.0 Hz), 17.3 (d, $J$ = 16.0 Hz) ppm; IR (ATR) 3229, 2988, 1668, 1258, 1187, 1019 cm$^{-1}$. HRMS (TOF-ESI) calcd for $C_{19}H_{25}NO_5P_2Na$ $[M + Na]^+$ 410.1286, found 410.1285. [a]Overlapping signals of two diastereotopic Et groups ($O\underline{CH_2}CH_3$). [b]Diastereotopic Et group ($OCH_2\underline{CH_3}$). [c]Signals of two diastereotopic Ph groups. [d]Diastereotopic Et group ($O\underline{CH_2}CH_3$).

**Diethyl 1-(*N*-acetylamino)-1-(diphenylphosphinoyl)ethylphosphonate (8g).** Colourless oil. $^{1}H$ NMR (400 MHz, $CDCl_3$) $\delta$ 8.10-7.97 (m, 4H) and 7.58-7.48 (m, 6H), 6.80 (br s, 1H), 4.18-3.96 (m, 4H)[a], 1.98 (dd, $J_1$ = 16.4, $J_2$ = 16.8 Hz, 3H), 1.96 (s, 3H), 1.21 (t, $J$ = 7.0 Hz, 3H)[b] and 1.18 (t, $J$ = 7.0 Hz, 3H)[b] ppm; $^{13}C$ NMR (100 MHz, $CDCl_3$) $\delta$ 170.0, [132.9 (d, $J$ = 9.2 Hz), 132.7 (d, $J$ = 9.2 Hz), 132.41 (d, $J$ = 3.0 Hz), 132.39 (d, $J$ = 1.5 Hz), 129.1 (d, $J$ = 97.9 Hz), 128.6 (d, $J$ = 81.2 Hz), 128.4 (d, $J$ = 12.3 Hz), 128.2 (d, $J$ = 12.2 Hz)][c], 63.5 (d, $J$ = 6.8 Hz), 59.4 (dd, $J_1$ = 63.3, $J_2$ = 147.6 Hz), 24.7, 18.2 (d, $J$ = 1.5 Hz), 16.3 (d, $J$ = 5.5 Hz)[b] and 16.2 (d, $J$ = 6.0 Hz)[b] ppm; $^{31}P$ NMR (162 MHz, $CDCl_3$) $\delta$ 35.3 (d, $J$ = 18.5 Hz), 20.8 (d, $J$ = 18.5 Hz) ppm; IR (ATR) 3429, 1690, 1237, 1181, 1020 $cm^{-1}$. HRMS (TOF-ESI) calcd for $C_{20}H_{27}NO_5P_2Na$ $[M + Na]^+$ 446.1262, found 446.1254. [a]Overlapping signals of two diastereotopic Et groups (O$\underline{CH_2}$$CH_3$). [b]Diastereotopic Et group ($OCH_2$$\underline{CH_3}$). [c]Signals of two diastereotopic Ph groups.

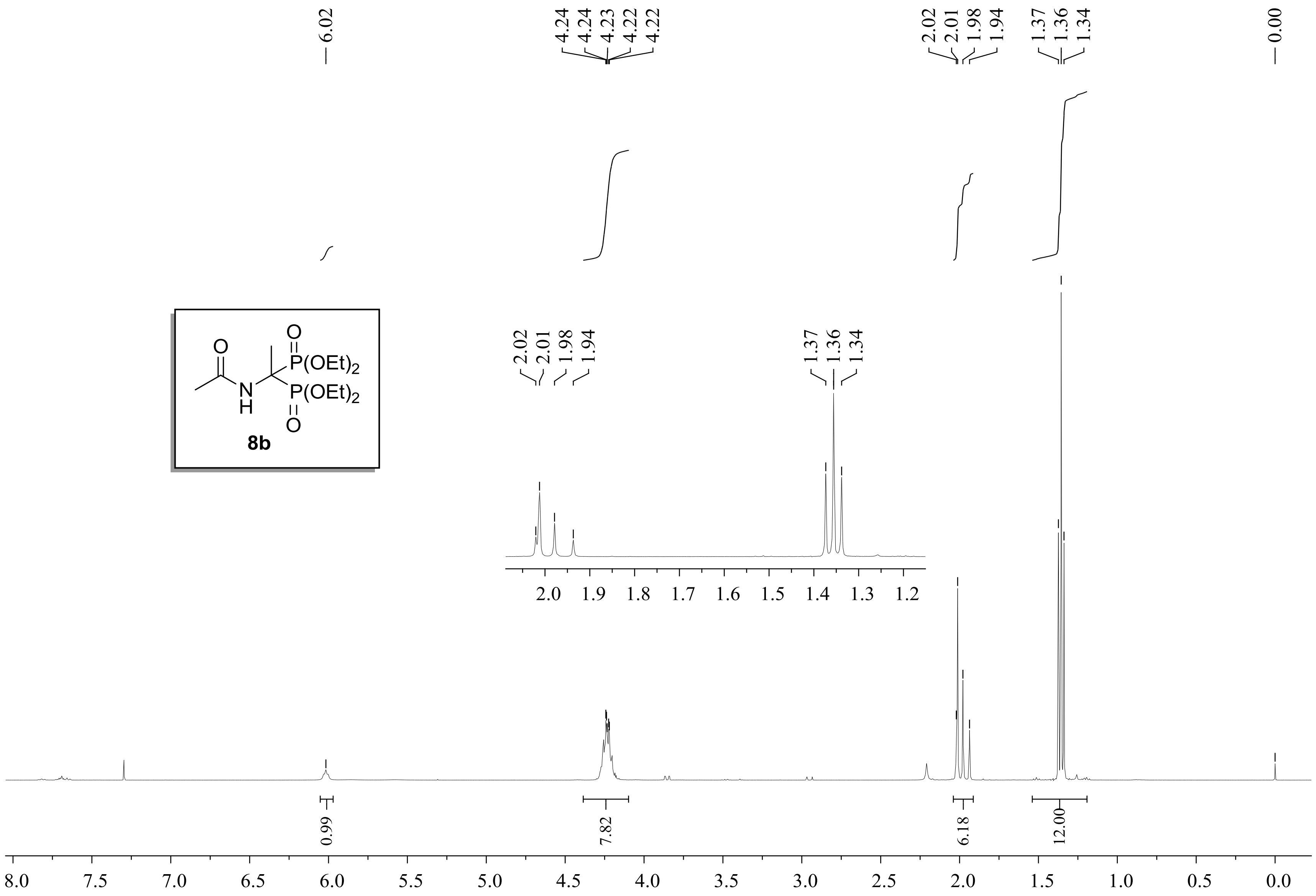


$^{1}$H NMR spectrum of tetraethyl 1-(*N*-acetylamino)ethylidenebisphosphonate (**8b**); 400 MHz/$CDCl_3$/TMS; δ (ppm).

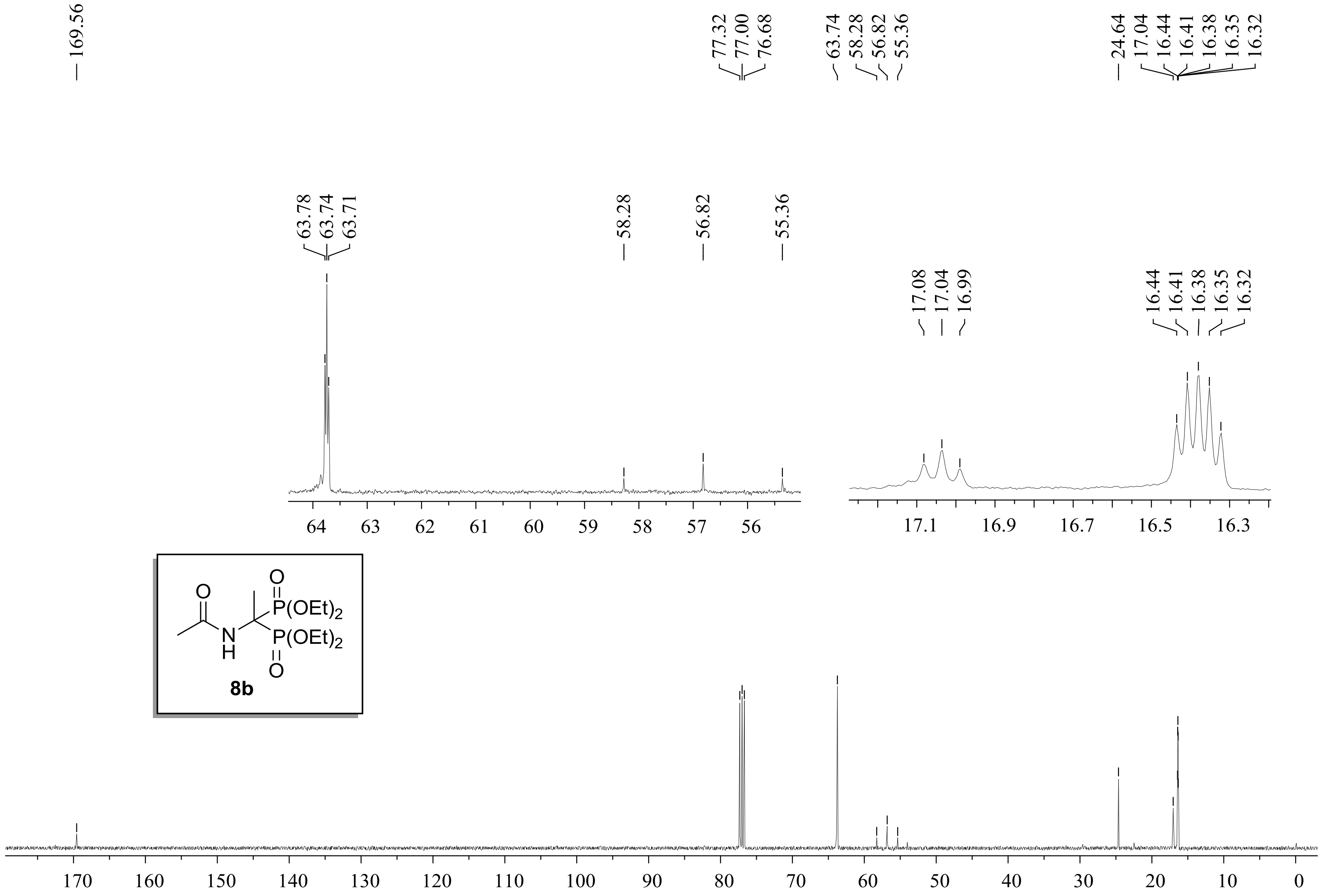


$^{13}$C NMR spectrum of tetraethyl 1-(*N*-acetylamino)ethylidenebisphosphonate (**8b**); 100 MHz/$CDCl_3$/TMS; δ (ppm).

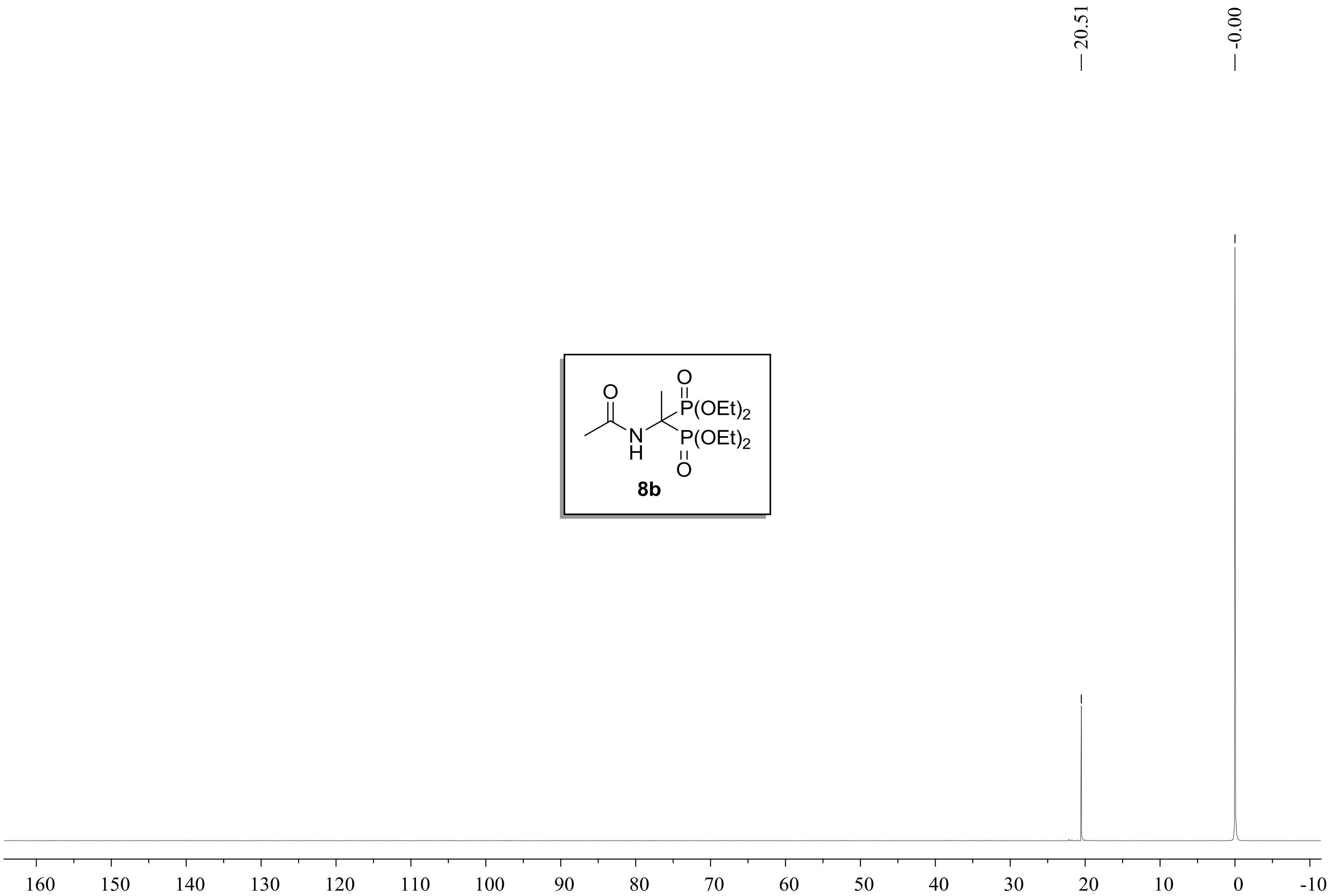


$^{31}$P NMR spectrum of tetraethyl 1-(*N*-acetylamino)ethylidenebisphosphonate (**8b**); 162 MHz/$CDCl_3$/$H_3PO_4$ (80%); δ (ppm).

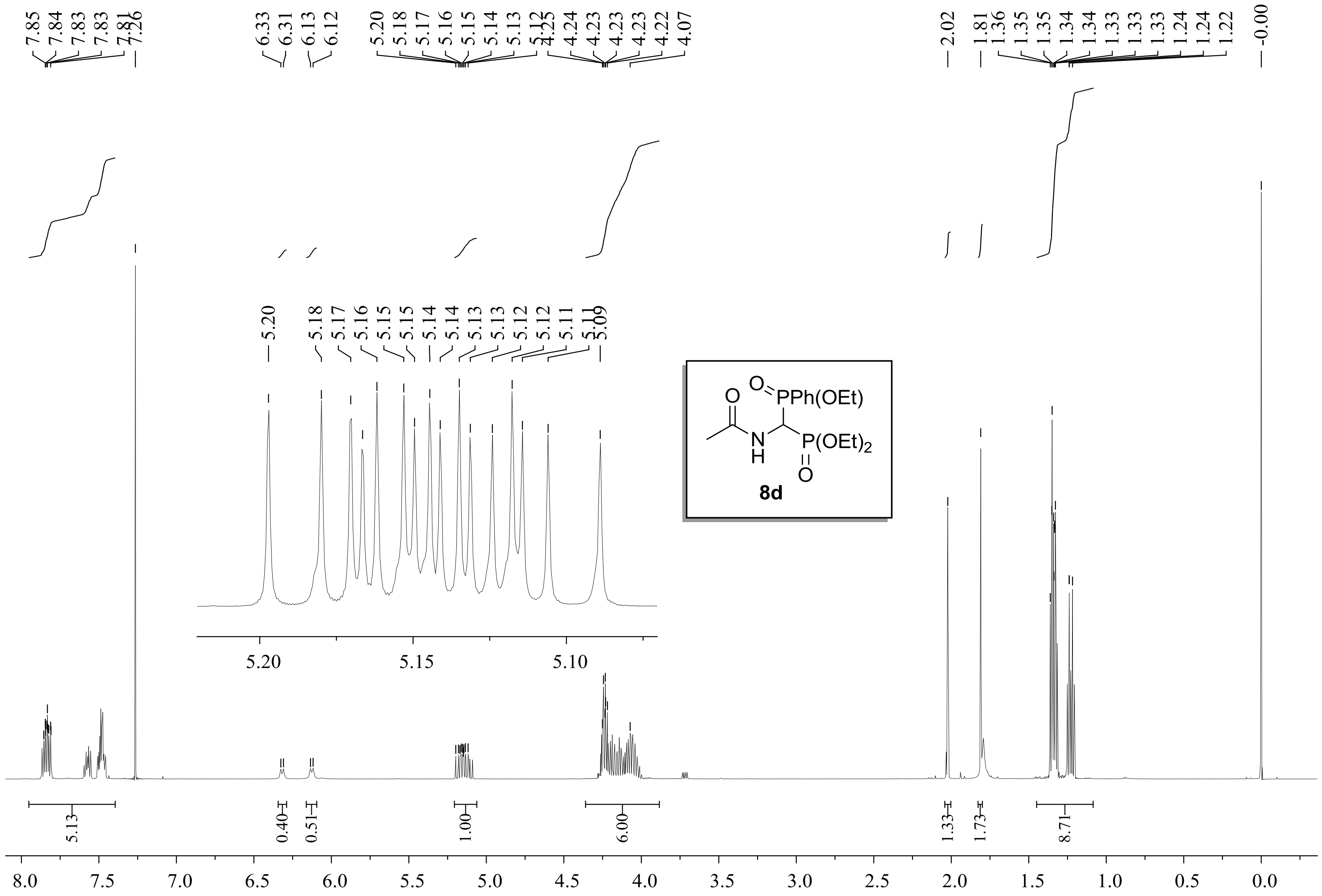

$^{1}$H NMR spectrum of the mixture of diastereomers of diethyl 1-(*N*-acetylamino)-1-[ethoxy(phenylphosphinyl)]methylphosphonate (**8d**); 600 MHz/$CDCl_3$/TMS; δ (ppm).

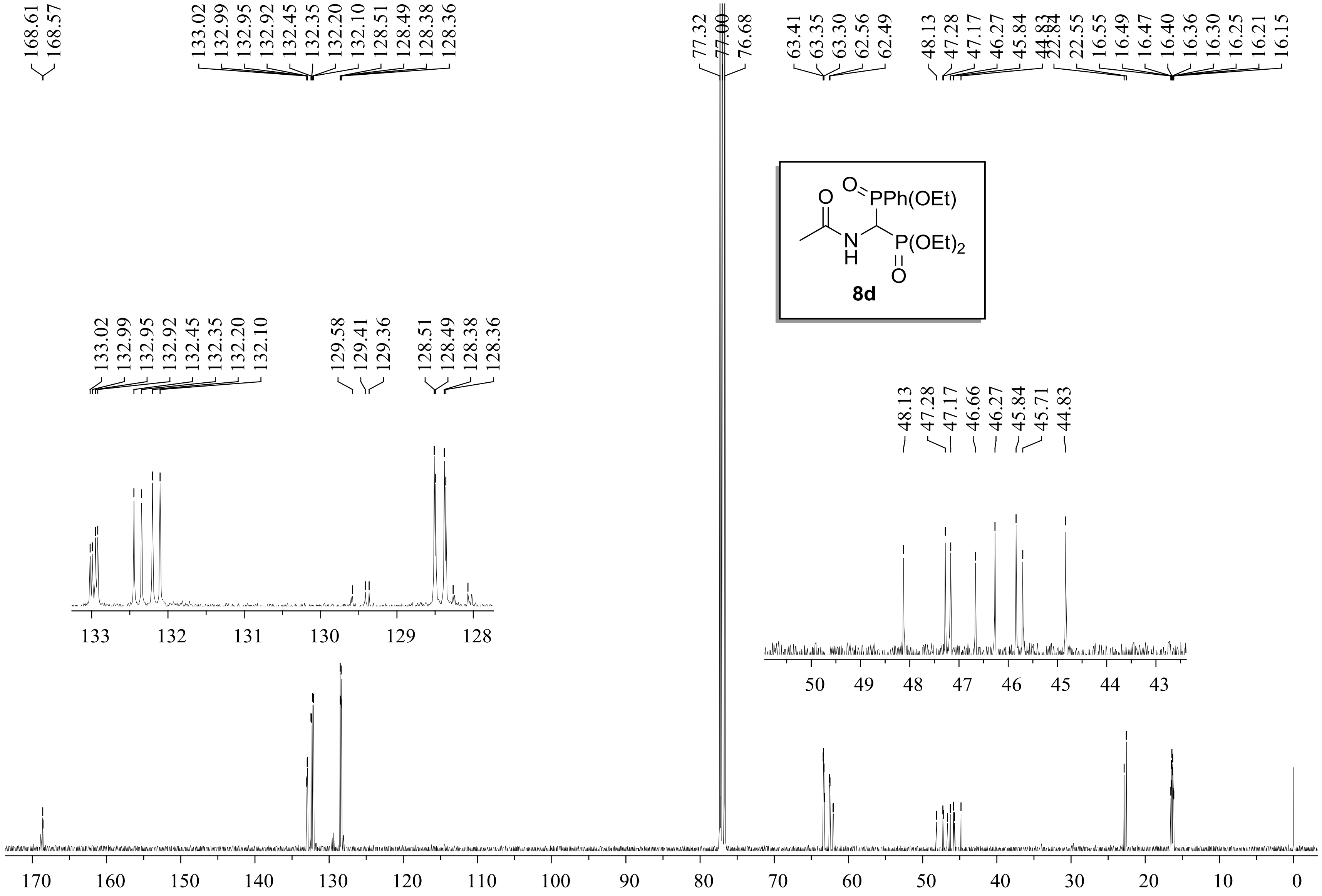

$^{13}$C NMR spectrum of the mixture of diastereomers of diethyl 1-(*N*-acetylamino)-1-[ethoxy(phenylphosphinyl)]methylphosphonate (**8d**); 100 MHz/$CDCl_3$/TMS; δ (ppm).

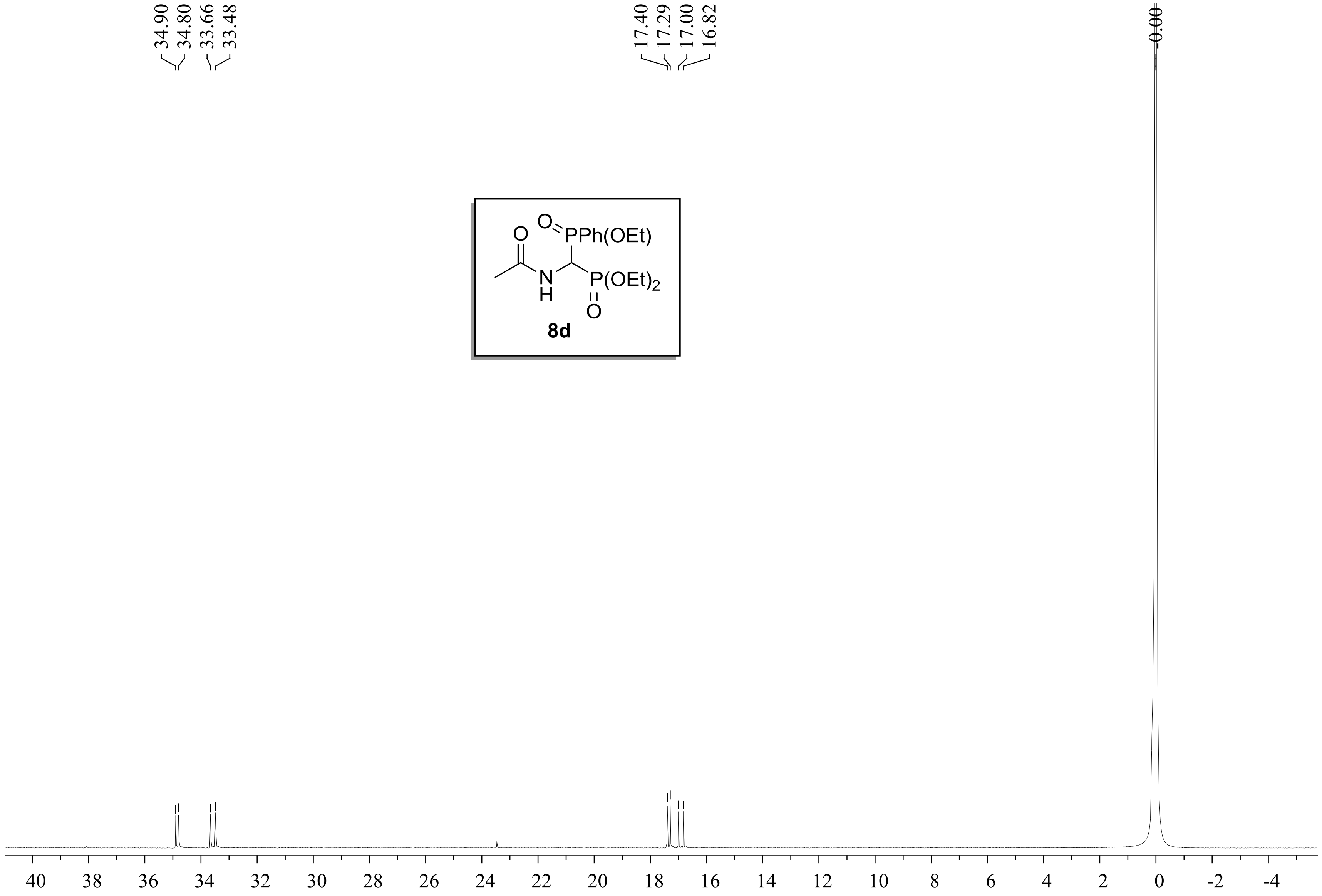


$^{31}$P NMR spectrum of the mixture of diastereomers of diethyl 1-(*N*-acetylamino)-1-[ethoxy(phenylphosphinyl)]methylphosphonate (**8d**); 162 MHz/$CDCl_3$; δ (ppm).

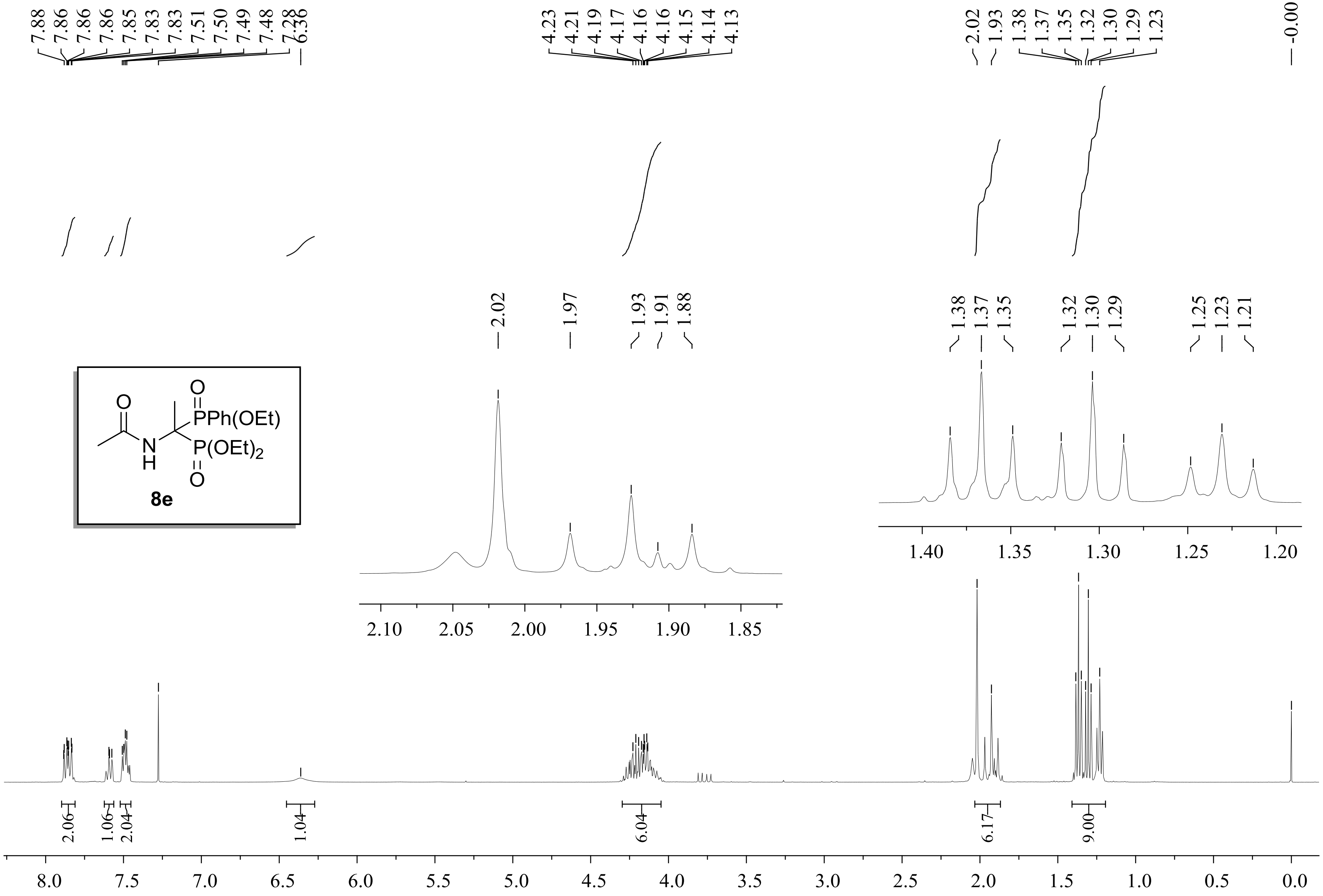

$^{1}$H NMR spectrum of one of the diastereomers of diethyl 1-(*N*-acetylamino)-1-[ethoxy(phenylphosphinyl)]ethylphosphonate (**8e**); 400 MHz/$CDCl_3$/TMS; δ (ppm).

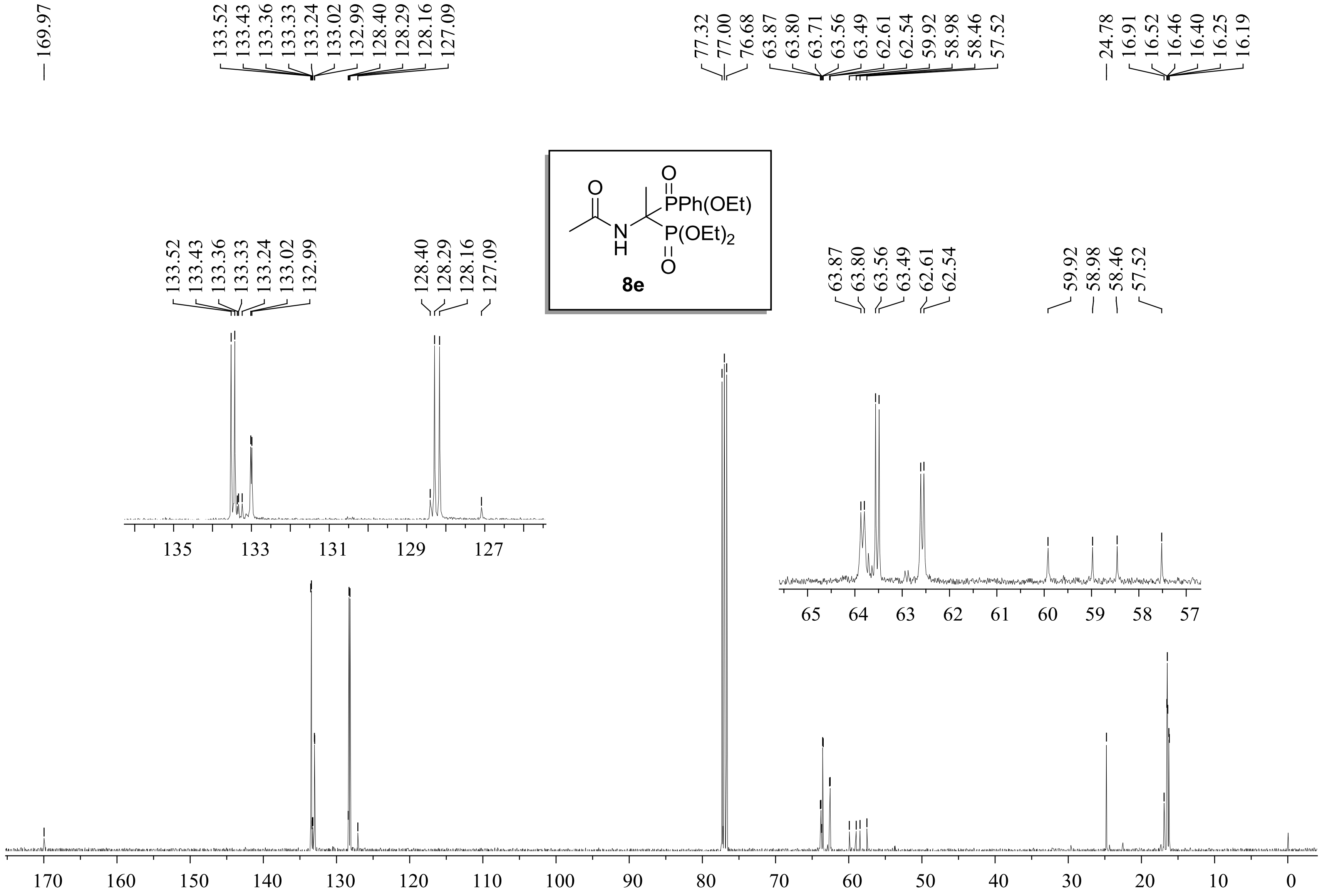


$^{13}$C NMR spectrum of one of the diastereomers of diethyl 1-(*N*-acetylamino)-1-[ethoxy(phenylphosphinyl)]ethylphosphonate (**8e**); 100 MHz/$CDCl_3$/TMS; δ (ppm).

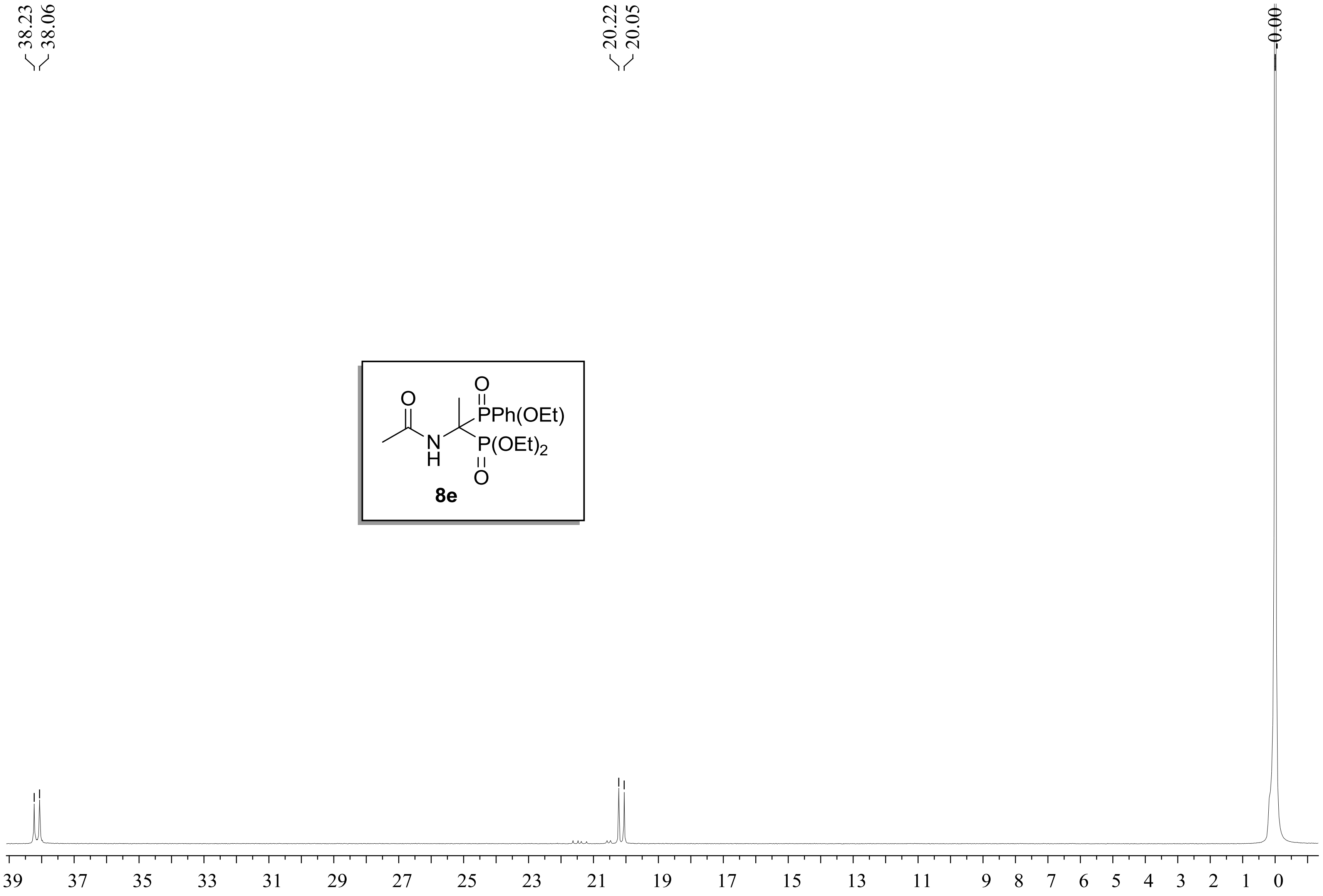


$^{31}P$ NMR spectrum of one of the diastereomers of diethyl 1-(*N*-acetylamino)-1-[ethoxy(phenylphosphinyl)]ethylphosphonate (**8e**); 162 MHz/$CDCl_3$; δ (ppm).

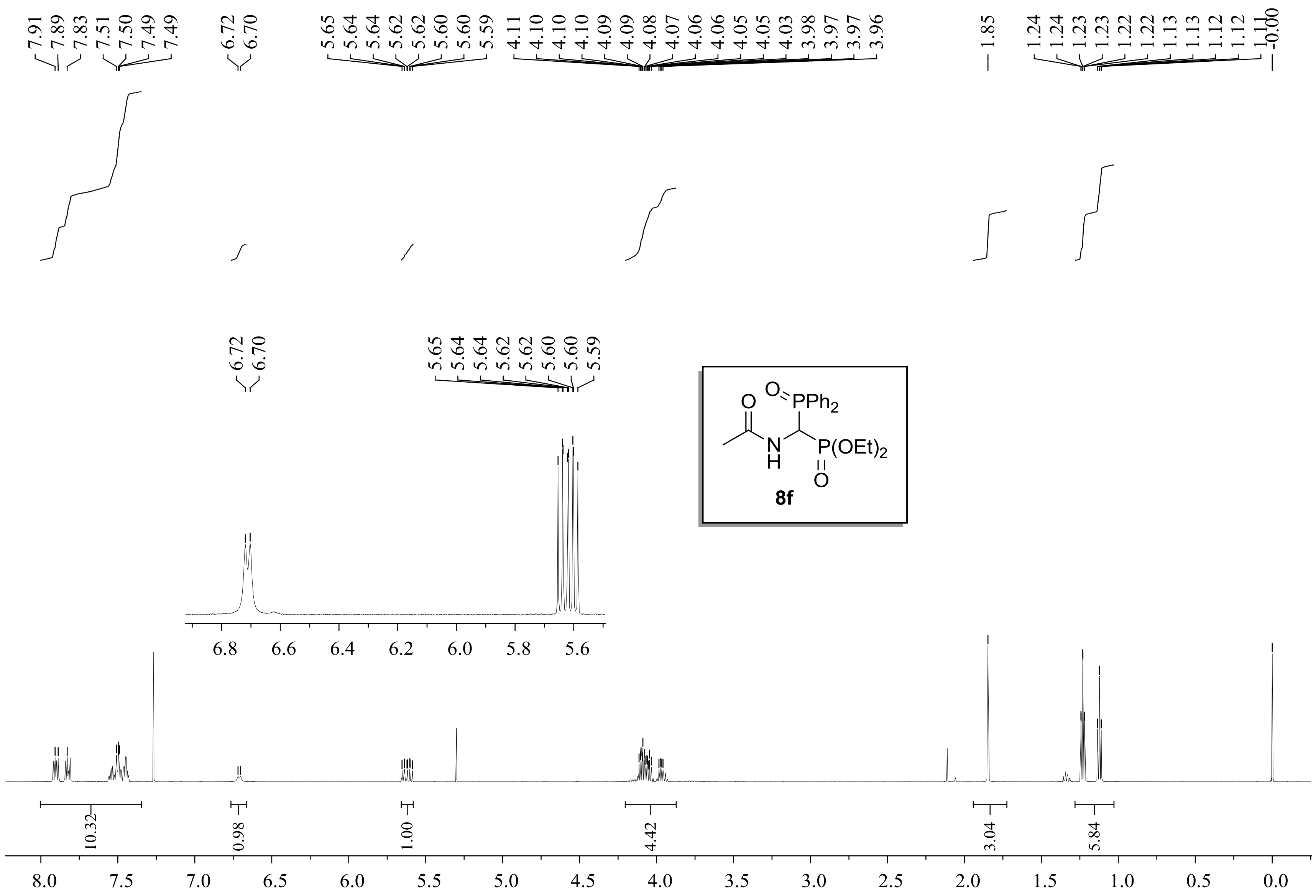

$^{1}$H NMR spectrum of diethyl 1-(*N*-acetylamino)-1-(diphenylphosphinoyl)methylphosphonate (**8f**); 600 MHz/$CDCl_3$/TMS; δ (ppm).

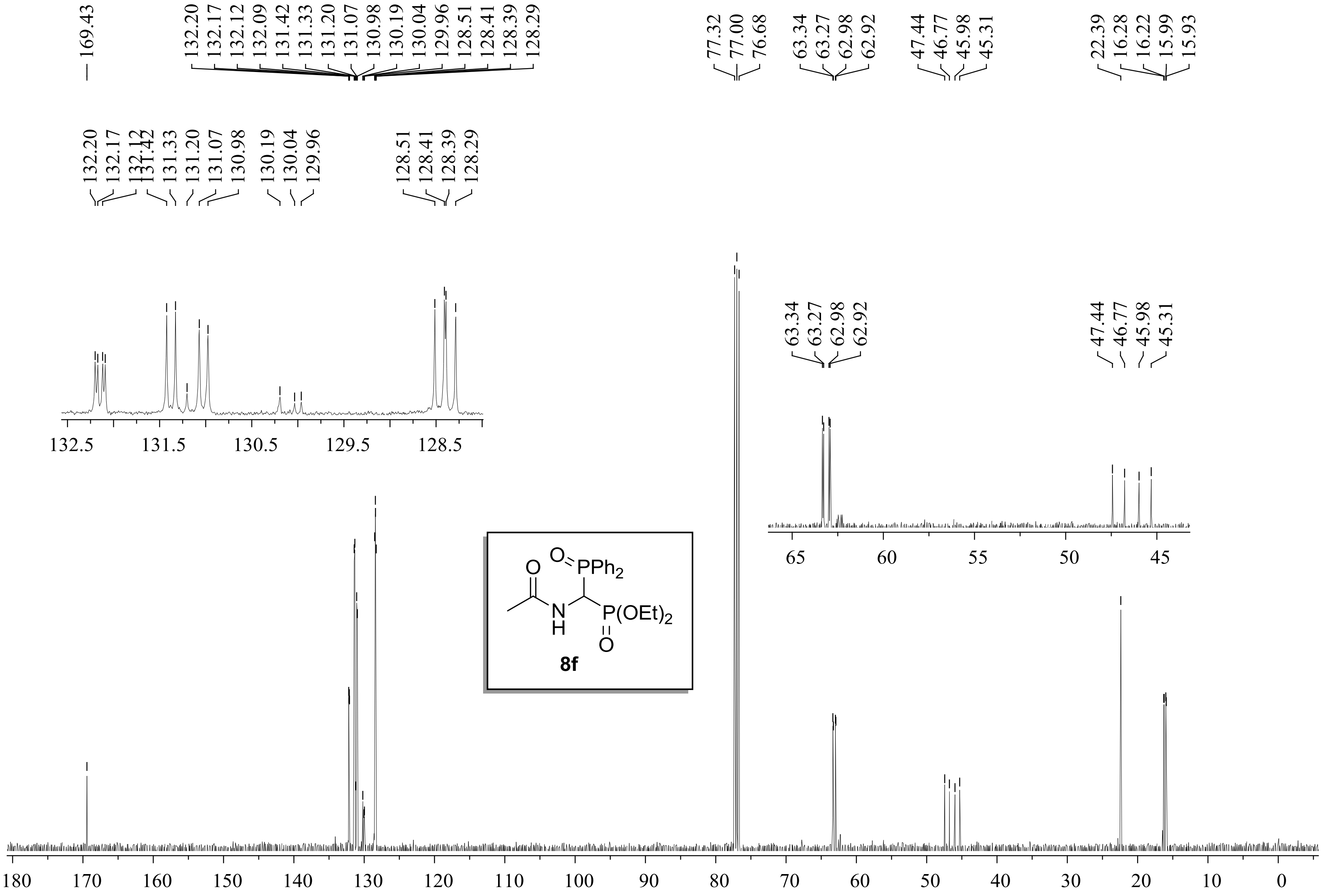


$^{13}$C NMR spectrum of diethyl 1-(*N*-acetylamino)-1-(diphenylphosphinoyl)methylphosphonate (**8f**); 100 MHz/$CDCl_3$/TMS; δ (ppm).

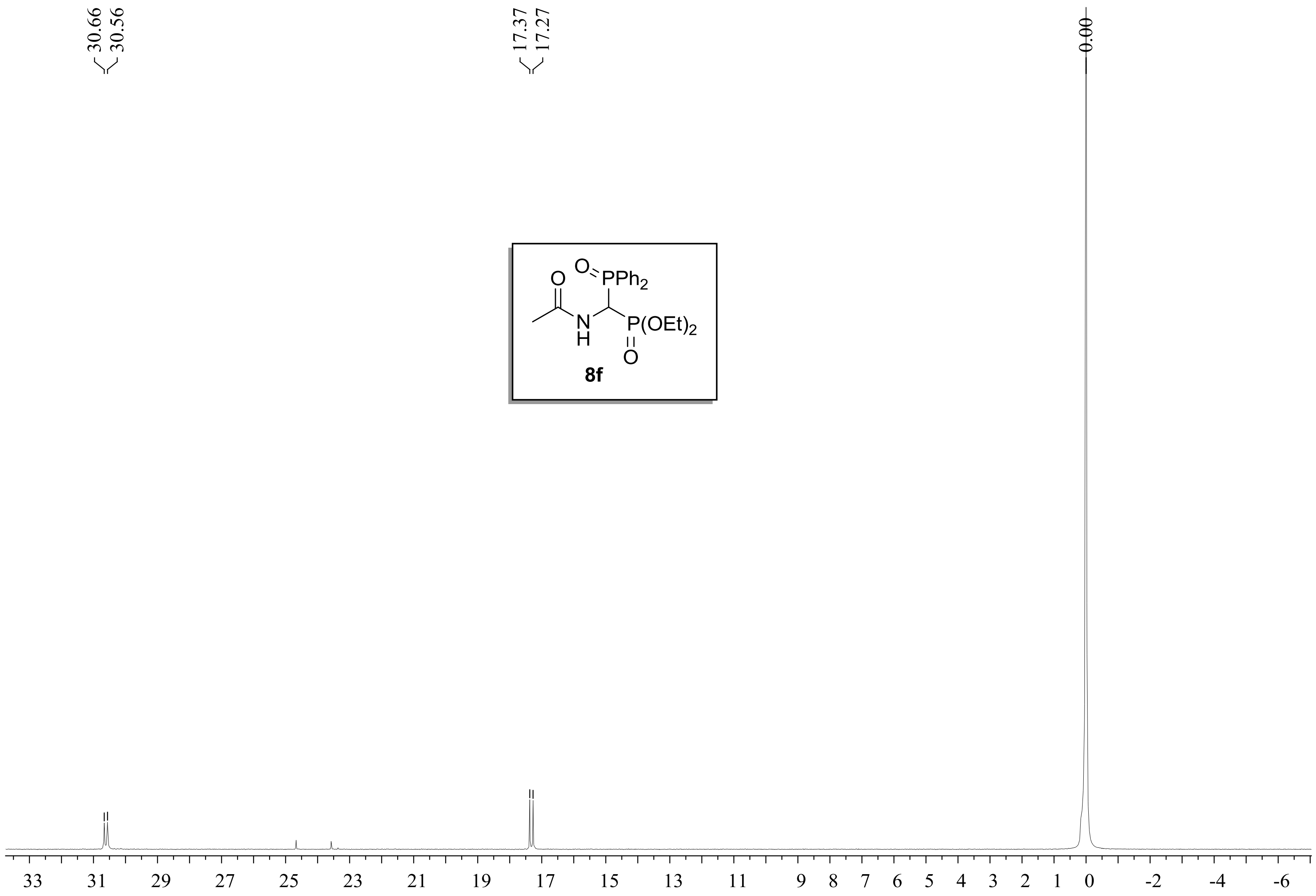


$^{31}$P NMR spectrum of diethyl 1-(*N*-acetylamino)-1-(diphenylphosphinoyl)methylphosphonate (**8f**); 162 MHz/$CDCl_3$; δ (ppm).
